# Supplementary material for: NF-κB Signaling Is Regulated by Fucosylation in Metastatic Breast Cancer Cells
Source: Biomedicines. 2020 Dec 12;8(12):600. doi: 10.3390/biomedicines8120600 (PMC7763959; doi:10.3390/biomedicines8120600)
Supplement: Supplementary file 1 [file biomedicines-08-00600-s001.zip › biomedicines-988207 supplementary/biomedicines-988207-proofreading-supp/supplementary file 2.pdf]

Supplemental Information

Experimental Methods

Reagents

**Antibodies and Lectin**		
**Item**	Manufacturer	Catalog Number
phospho-NF-κB	Cell Signaling	3033
NF-κB	Cell Signaling	8242
IκBα	Cell Signaling	4814
Bcl10	Cell Signaling	4237
Tollip	BioRad	MCA6034
β-tubulin	Santa Cruz	Sc-55529
Aleuria aurantia lectin	Vector Labs	B-1395
**Real-Time PCR**		
**Item**	Manufacturer	Catalog Number
Tnfa	Thermo Fisher Scientific	*Mm00443258_m1*
Icam1	Thermo Fisher Scientific	*Mm00516023_m1*
Hprt	Thermo Fisher Scientific	*Mm01545399_m1*
Tbp	Thermo Fisher Scientific	*Mm00446973_m1*
Tollip	BioRad	*qMmuCED0047589*
Bcl10	BioRad	*qMmuCIP0031559*
Gapdh	*Forward-GCACAGTCAAGGCCGAGAAT*	*Reverse-GCCTTCTCCATGGTGGTGAA*

Cell preparation

Cells were lysed in 8 M urea, 50 mM Tris-HCl, pH 8.5. Samples were sonicated in a Bioruptor® sonication system from Diagende Inc. (30 sec/30 sec on/off cycles for 15 minutes, 4 °C). Following centrifugation at 12,000 rpm for 15 minutes, protein concentrations were determined using a Bradford protein assay kit (cat. num. 5000002, Bio-Rad). Protein samples in equal amounts (50 μg) were reduced with 5 mM tris(2-carboxyethyl)phosphine hydrochloride (TCEP) and alkylated with 10 mM chloroacetaminde (CAM). Samples were diluted with 100 mM Tris-HCl to a final urea concentration of 2 M and digested overnight with Trypsin/Lys-C Mix Mass Spectrometry (1:100 protease/substrate ratio, cat. num. V5072, Promega) [43-45].

Peptide purification and labeling

Peptides were desalted on 50 mg Sep-Pak® Vac (Waters Corporation) employing a vacuum manifold. After elution from the column in 70% acetonitrile (ACN)–0.1% formic acid (FA), peptides were dried by speed vacuum and resuspended in 24 μL of 50 mM triethylammonium bicarbonate (TEAB). Peptide concentration was measured using Pierce Quantitative Colorimetric Peptide Assay Kit (cat. num. 23275, Thermo Fisher Scientific) to ensure that an equal amount of each sample was labeled. Samples were then Tandem Mass Tag (TMT)-labeled with 0.2 mg of reagent resuspended in 20 μL acetonitrile for 2 hours at room temperature (**Figure 1B**, cat. num. 90309, Thermo Fisher Scientific TMT10plex™ Isobaric Label Reagent Set; lot no. UH285567 and 131C lot UD280157A). The labelling reactions were quenched with hydroxylamine at room temperature 15 minutes. The labelled peptides were then mixed and dried by speed vacuum.

High-pH basic fractionation

The peptide mixture was resuspended in 0.1% TFA (trifluoroacetic acid), and 150 μg was fractionated on Pierce™ High-pH reversed-phase peptide fractionation spin columns following the manufacturer's instructions (cat. num. 84868). Each fraction was dried by speed vacuum and resuspended in 24 μL 0.1% FA.

Nano-LC–MS/MS Analysis

Nano-LC–MS/MS analyses were performed on an EASY-nLC™ HPLC system coupled to an Orbitrap Fusion™ Lumos™ mass spectrometer (Thermo Fisher Scientific). One-third of each fraction was loaded onto a reversed-phase PepMap™ RSLC C18 column with an Easy-Spray tip at 400 nL/min (ES802A, 2 μm, 100 Å, 75 μm x 25 cm). The peptides were eluted using 4%–28% B over 160 minutes, 28%–35% B over 5 mins, 35%–50% B for 14 minutes, dropping from 50% to 10% B over the final 1 min (mobile phases A: 0.1% FA, water; B: 0.1% FA, 80% acetonitrile). Mass spectrometer settings included capillary temperature of 275 °C and ion spray voltage at 2.5 kV. The mass spectrometer method was operated in positive-ion mode with

a 4 second cycle time data-dependent acquisition with advanced peak determination and Easy-IC on (internal calibrant). Precursor scans (m/z 400-1750) were done with an orbitrap resolution of 120,000, 30% RF lens, 50 ms maximum inject time (IT), standard automatic gain control (AGC) target, including charges of 2 to 6 for fragmentation with 60 s dynamic exclusion. Higher-energy collisional dissociation (HCD) MS2 scans were performed at 50,000 orbitrap resolution, fixed collision energy of 35%, 20% normalized AGC target, and dynamic maximum IT.

Data analysis

The resulting RAW files were analyzed in Proteome Discover™ 2.4 (Thermo Fisher Scientific) with FASTA databases including Swiss-Prot UniProt *Mus musculus* sequences plus common contaminants. Quantification methods utilized isotopic impurity levels available from Thermo Fisher. SEQUEST HT searches were conducted with a maximum number of 2 missed cleavages, precursor mass tolerance of 10 ppm, and a fragment mass tolerance of 0.02 Da. Static modifications used for the search were: 1) carbamidomethylation on cysteine (C) residues; 2) TMT sixplex label on lysine (K) residues and the N-termini of peptides. Dynamic modifications used for the search were oxidation of methionines and acetylation of N-termini. Percolator False Discovery Rate was set to a strict setting of 0.01 and a relaxed setting of 0.05. Values from both unique and razor peptides were used for quantification. In the consensus workflow, peptides were normalized by total peptide amount with no scaling. Resulting grouped abundance values for each sample type, abundance ratio values, and respective p-values (t-test) from Proteome Discoverer™ were exported to Microsoft Excel and are available as supplemental files. The mass spectrometry proteomics data have been deposited to the ProteomeXchange Consortium via the PRIDE partner repository with the dataset identifier PXD021413 and 10.6019/PXD021413 [47].

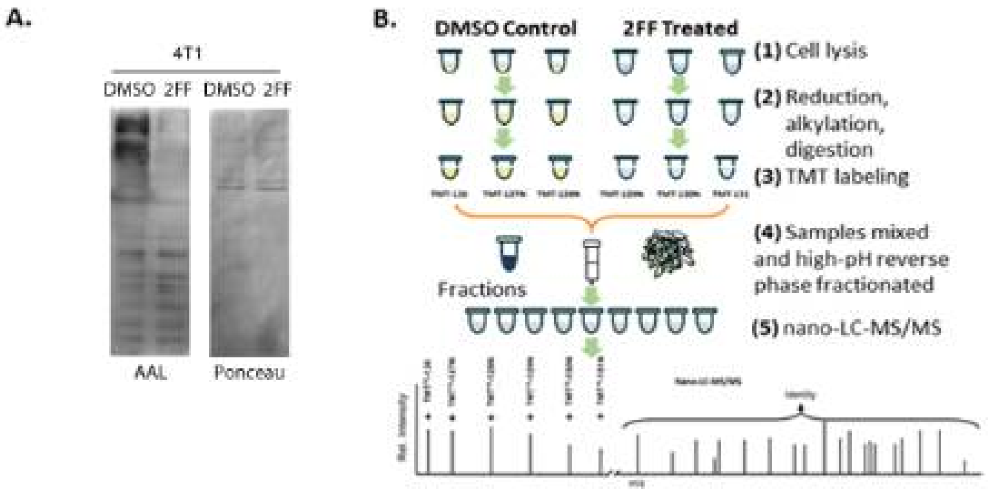

Supplemental Figure 1. Global proteomic analysis of 4T1 cells treated with a fucosylation inhibitor. (A) *Aleuria aurantia* lectin (AAL) blot analysis of DMSO- versus 2FF- (500 μM) treated lysates. AAL was used to detect fucosylation. Predominant bands in Ponceau staining show loading. **(B)** Schematic of the LC–MS protocol.

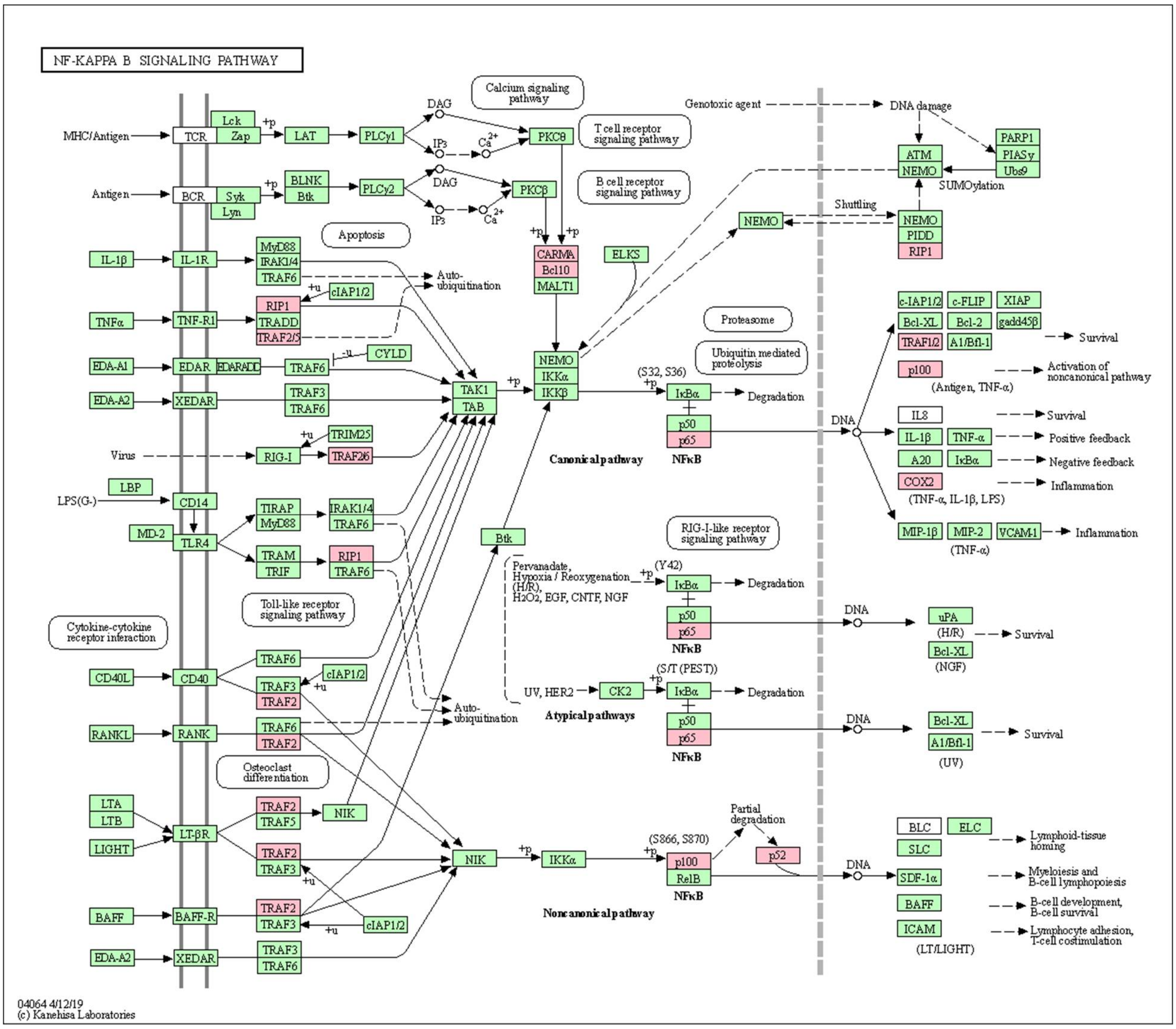

Supplemental Figure 2. KEGG NF-KAPPA B signaling pathway. Proteins in red were significantly decreased in 2FF-treated cells vs. DMSO-treated cells.

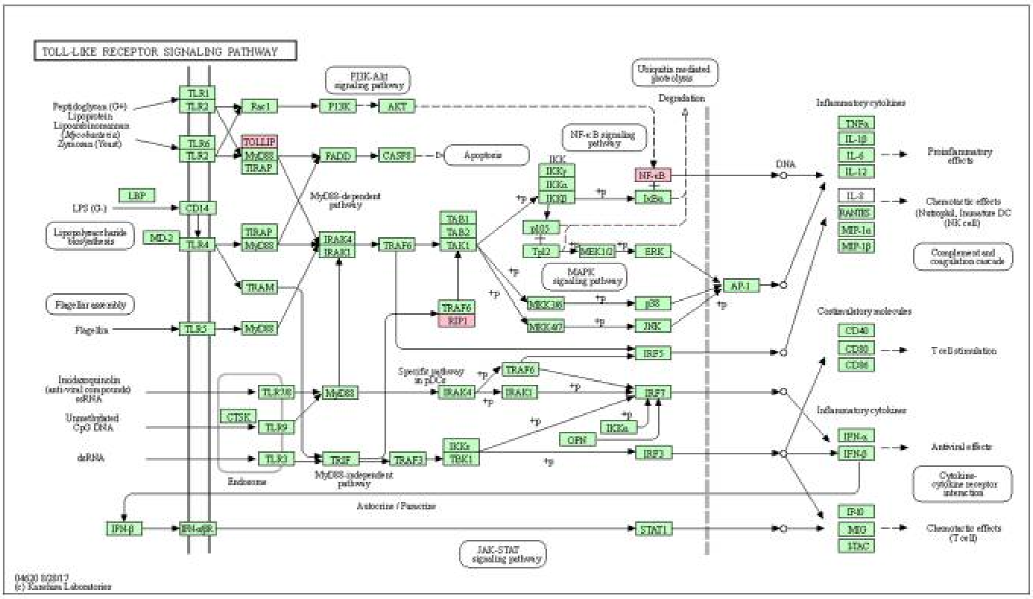

Supplemental Figure 3. KEGG toll-like receptor signaling pathway. Proteins in red were significantly decreased in 2FF- vs. DMSO-treated cells.

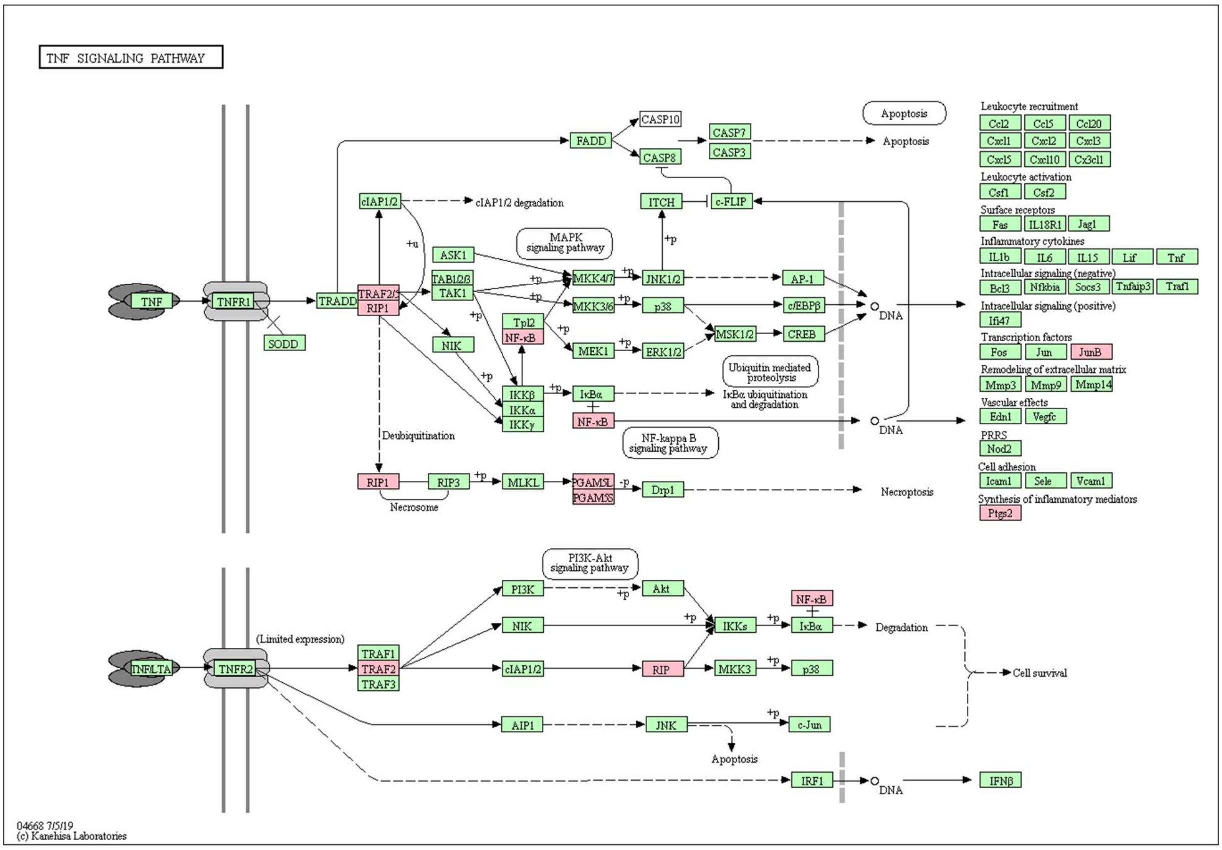

Supplemental Figure 4. KEGG TNF signaling pathway. Proteins in red were significantly decreased after 2FF treatment.
